# Poly-(Lactic-co-Glycolic) Acid Nanoparticles for Synergistic Delivery of Epirubicin and Paclitaxel to Human Lung Cancer Cells

**DOI:** 10.3390/molecules25184243

**Published:** 2020-09-16

**Authors:** Nikita Sharma, R. Mankamna Kumari, Nidhi Gupta, Asad Syed, Ali H. Bahkali, Surendra Nimesh

**Affiliations:** 1 Department of Biotechnology, School of Life Sciences, Central University of Rajasthan, Ajmer 305817, India; 2015phdbt01@curaj.ac.in (N.S.); 2014phdbt005@curaj.ac.in (R.M.K.); 2 Department of Biotechnology, IIS (Deemed to be University), Jaipur Rajasthan 302020, India; nidhi.gupta@iisuniv.ac.in; 3 Department of Botany and Microbiology, College of Science, King Saud University, P.O. Box 2455, Riyadh 11451, Saudi Arabia; assyed@ksu.edu.sa (A.S.); abahkali@ksu.edu.sa (A.H.B.)

**Keywords:** PLGA nanoparticles, epirubicin, paclitaxel, MTT, p53

## Abstract

Combination therapy using chemically distinct drugs has appeared as one of the promising strategies to improve anticancer treatment efficiency. In the present investigation, poly-(lactic-co-glycolic) acid (PLGA) nanoparticles electrostatically conjugated with polyethylenimine (PEI)-based co-delivery system for epirubicin and paclitaxel (PLGA-PEI-EPI-PTX NPs) has been developed. The PLGA-PEI-EPI-PTX NPs exhibited a monodispersed size distribution with an average size of 240.93 ± 12.70 nm as measured through DLS and 70.8–145 nm using AFM. The zeta potential of 41.95 ± 0.65 mV from −17.45 ± 2.15 mV further confirmed the colloidal stability and PEI modification on PLGA nanoparticles. Encapsulation and loading efficiency along with in vitro release of drug for nanoparticles were done spectrophotometrically. The FTIR analysis of PLGA-PEI-EPI-PTX NPs revealed the involvement of amide moiety between polymer PLGA and PEI. The effect of nanoparticles on the cell migration was also corroborated through wound healing assay. The MTT assay demonstrated that PLGA-PEI-EPI-PTX NPs exhibited considerable anticancer potential as compared to the naïve drugs. Further, p53 protein expression analysed through western blot showed enhanced expression. This study suggests that combination therapy using PLGA-PEI-EPI-PTX NPs represent a potential approach and could offer clinical benefits in the future for lung cancer patients.

## 1. Introduction

Lung cancer is one of the leading causes of death, contributing to 18.4% of total cancer deaths, followed by female breast cancer (11.6%), prostate cancer (7.1%) and colorectal cancer (6.1%) [1]. Histologically, it is classified into non-small-cell lung carcinoma (80–85%), and small-cell lung carcinoma (15–20%) with a 5 year survival rate of 5% [1]. Surgical resection and chemotherapy are the most effective approaches for the treatment of cancer. While progress has been made in the chemotherapy and radiation therapy approaches globally, the survival rate among patients remains unchanged [2]. Although many conventional chemotherapeutic drugs have been used for treatment, long term use results in the development of resistance among the patients. To overcome these issues, several strategies have been developed that includes the inhibition of efflux pump activity through specific molecules, or design of novel therapeutic agents which are less susceptible to drug resistance, or combination of two or more pharmaceutical agents that targets different signaling pathways in cancer cells [3]. Combination therapy is generally preferred as it not only increases response rates but also reduces the occurrence of resistance among patients, which is often associated with monotherapy [4]. This can further delay cancer adaptation and reduce the possibility of cancer progression.

Anticancer drugs such as paclitaxel (PTX) and epirubicin (EPI) are administered as first line treatments in cancer therapy. Epirubicin is the 4′ epi-isomer of doxorubicin and belongs to the anthracycline drug class. It is widely used as a chemotherapeutic agent against several cancers including breast, colon, throat, bladder, acute leukemia and lung cancer. Though its exact mechanism of action is not known, but it inhibits DNA and RNA synthesis by intercalating between the base pairs of DNA. Studies revealed that it acts as a topoisomerase II inhibitor and also upregulates the expression of reactive oxygen species (ROS) [5]. Acute dose toxicity of EPI results in myelosuppression, which consequently decreases the red and white blood cells and platelet count in the blood. Additionally, it can cause transient cardiac arrhythmia and changes in the electrocardiogram [6]. These aforementioned side effects are associated with poor distribution of EPI among the tissues of healthy and cancerous cells.

Another anticancer drug, paclitaxel extracted from the bark of *Taxus brevifolia*, is an effective chemotherapeutic drug against lung, breast, and ovarian cancer. It acts by stabilizing the microtubules and inhibiting the G2/M phase of cell cycle, thereby causing cell death. The major limitation associated with the use is its low water solubility [7], so formulations which improve their pharmacokinetics and solubility are imperative for their translational use in clinics. Even though combinational approach targets various signaling routes, it is extremely difficult to monitor their pharmacokinetics, bio-distribution or cellular uptake within the tumor cells and to make them act synergistically.

To circumvent these therapeutic challenges, an ideal nano-sized packaging system needs to be developed that targets the drug candidates to the cancerous sites, thereby decreasing the toxicity and escalating the therapeutic efficiency of the respective drugs. Additionally, leaky vasculature in the tumor microenvironment results in enhanced permeability retention (EPR) effect which further facilitates the preferential accumulation of nanocarriers to tumorous site(s), and limiting drug distribution in healthy cells [8]. Thus, various nano-based cargos have been explored for the delivery of therapeutic molecules to their target sites [9].

Several polymeric nanoparticles have been explored for their delivery to the particular site in a safe and efficient manner. Among various synthetic polymers, PLGA is a FDA approved polymer which has tremendous potential as a drug delivery vehicle owing to its biodegradability, bioavailability, biocompatibility, ease of surface modifications according to the site of drug release [10]. At acidic pH, PLGA undergoes hydrolysis of ester bond resulting in the degradation of the polymer into their respective monomeric units of lactic acid and glycolic acid. These monomeric units can further enter into the glycolysis and Kreb’s cycle, thereby avoiding long term toxicity concerns [11]. The encapsulation of anticancer drugs within the particle at nanometer scale facilitates enhanced release rate with reduce risk of toxicity due to large surface area by volume ratio and target specific delivery. Previous studies have depicted that PLGA nanoparticles formulated with polyethylenimine (PEI) resulted in higher cellular uptake, sustained siRNA delivery and efficient gene delivery [12].

Moreover, plethora of studies has been done to explore the potential of nanocarriers against lung cancer. Recently, Lin et al., developed a dual ligand anti-CA IX (anti-carbonic anhydrase IX) and CPP33 functionalized liposomes encapsulating triptolide (dl-TPL-lip). The results showed an enhanced cytotoxicity of TPL in CA-IX having lung cancer cells, with prolonged sustained release of the drug. A remarkable tumor growth inhibiting activity and tumor penetration potential was demonstrated through 3D tumor spheroids. When administered through pulmonary route, dl-TPL-lip considerably increased the anticancer efficacy of TPL without any systemic toxicity in tumor bearing mice [13]. For a targeted delivery of drug, mesenchymal stem cells (MSC) were utilized. In the study, MSC was used as a carrier for the delivery of docetaxel (DTX)-loaded nanoparticles. Increased penetration activity of MSC was observed as compared to the fibroblast cells. Both in vitro and in vivo studies demonstrated the intercellular translocation of nanoparticles from MSC to tumor cells [14,15,16]. Additionally, to enhance the bioavailability of itraconazole (ITR), chitosan-coated PLGA nanoparticle was designed, synthesized and characterized and their anticancer potential was analyzed in H1299 NSC lung carcinoma cell. The cytotoxicity was increased in the nanoparticle treated cells as compared to the free drug. Also, formulated nanoparticles induced greater protein expression of Bax and p53 as compared to the individual ITR. The nanoparticles were more efficient in arresting the cells at different cell cycle phases [17]. The present work aims to develop a delivery vehicle for the synergistic effect of epirubicin and paclitaxel and to evaluate their anticancer potential against the A549 lung cancer cell lines. In the current study, it was hypothesized that the synergistic delivery of EPI and PTX via PLGA nanoparticles could be improved by introduction of positive charge onto the nanoparticles. To accomplish this hypothesis positive charge would be introduced using polycationic polymer PEI, so as to enhance the uptake of PLGA nanoparticles into lung cancer cells. The novelty of the proposed work lies in the introduction of a high positive charge in order to improve the uptake of nanoparticles thereby resulting in enhancement of drug uptake and subsequent anticancer efficacy. To accomplish this hypothesis, we synthesized PEI modified PLGA nanoparticles for the delivery of PTX and EPI with the aim to improve the efficiency of these anticancer drug in model cancer cells. The nanoparticles were physicochemically characterized in terms of size and zeta potential employing DLS, AFM and Zetasizer. Further, the chemical composition of the nanoparticles was monitored by FTIR studies. The entrapment and loading efficiency of both PTX and EPI was determined by preparation of several sets of nanoparticles formulations. The entrapped anticancer drugs release profile was investigated at two pHs i.e., 5.4 and 7.6. The in vitro uptake of the nanoparticles was monitored in lung adenocarcinoma cells, A549, followed by apoptosis using ethidium bromide (EtBr) and acridine orange staining. Further, the cell migration capability of A549 cells was studies using scratch assay. The anticancer potential of drug entrapped nanoparticles was determined employing MTT assay. Western blotting was employed to investigate the protein levels of apoptotic gene p53 in A549 cells.

## 2. Results and Discussion

### 2.1. Evaluation of Combination Ratio of EPI and PTX

#### 2.1.1. Determination of Cell Toxicity

**Figure 1:**
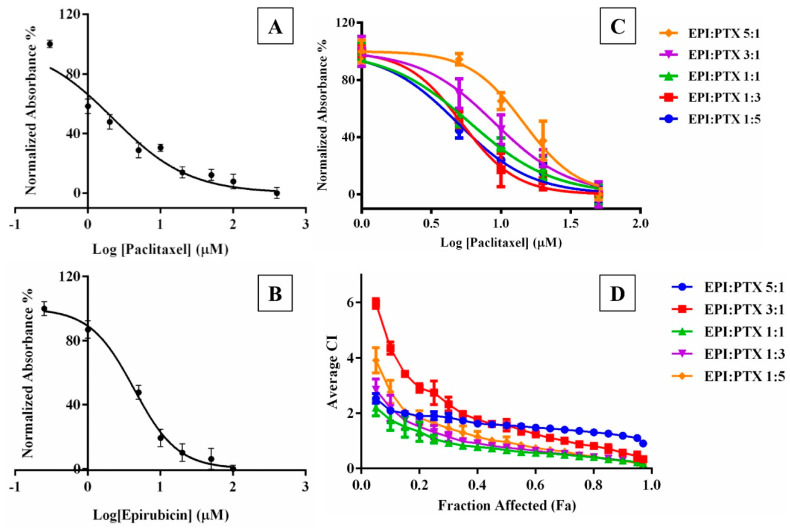
Dose response curve for (**A**) Paclitaxel and (**B**) Epirubicin in A549 cell line as a monotherapy as well as in combination (**C**) at 5 fixed ratios EPI:PTX (5:1, 3:1, 1:1, 1:3, 1:5), (**D**) Combination index (CI) values for the combination at different fraction affected (Fa) in cancer cells. Fa represents the fraction of cells that results in cell death by the drug combination. Data shown as mean ± SD (n = 3).

The cytotoxicity of EPI and PTX as individual monotherapies as well as in combination was determined in A549 cells (Figure 1). After 48 h of incubation, A549 cell line exhibited high sensitivity to PTX and EPI with IC_50_ value of 2.463 ± 2.159 µM and 2.134 ± 1.828 µM, respectively. Further, the toxicity of PTX and EPI in combination was assessed at five various molar ratios (EPI: PTX 5:1, 3:1, 1:1, 1:3, 1:5) for over 48 h duration. Figure 1 shows that PTX combined with EPI, showed a lower IC_50_ values as compared to PTX alone. Similar results were observed when PTX was combined with piperine on breast cancer cells such as MCF-7 cells [18]. At 1:5 constant ratios, both the isobologram analysis and combination index revealed the synergistic effect of PTX on model cancer cell lines.

#### 2.1.2. Determination of Combination Index Values

The cytotoxic effect of EPI and PTX in combination was investigated in A549 lung cancer cell lines, at varying molar ratios of EPI and PTX. As previously mentioned in the Chou and Talalay method, combination index (CI) values with CI < 1 reflects synergistic effect, CI = 1 shows additive effect and CI > 1 shows antagonistic effect [19]. The CI values were determined at different fraction of affected cells (Fa) extending from 0.1–0.9. For an effective anticancer effect, the higher values of Fa are considered for the treatment, as eradication of entire cancer cell is desirable.

The results showed an increased synergistic effect (for CI < 1) at higher Fa levels, at almost all EPI:PTX molar ratio combinations [20]. Furthermore, a molar ratio of 1:1 was analysed as optimum since it displayed synergistic effect (CI < 1) at all affected fractions (Fa) in the cell line (Figure 1). Similar results were obtained when this combination were administered in MCF-7 cells. It was shown that at 1:1 EPI/PTX, the cells showed a synergistic effect against breast cancer cell lines [21].

### 2.2. Preparation and Characterization of PLGA-PEI-EPI-PTX Nanoparticles

**Figure 2:**
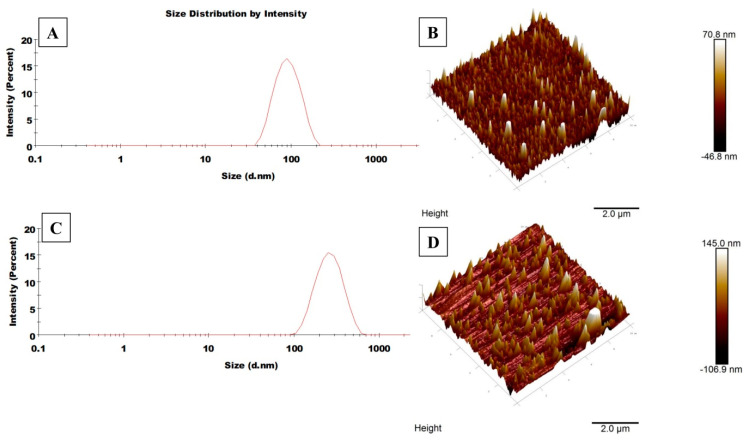
Illustrative DLS spectrum and AFM images of (**A**) DLS spectrum of void PLGA NPs here size is 95.5 nm, (**B**) AFM image of void PLGA NPs here average size is 70 nm, (**C**) DLS spectrum of PLGA-PEI-EPI-PTX NPs here size is 241.3 nm, (**D**) AFM image of PLGA-PEI-EPI-PTX NPs here average size is 140 nm.

In the present study, the nanoparticles were prepared using the solvent displacement method (or nanoprecipitation method) in which the hydrophobic PLGA polymer was dissolved in a partially water miscible solvent, acetone. This technique is preferred over several other methods including homogenisation, sonication etc., owing to its simple, easy and less energy-consuming approach. The presence of PF68 acts as a stabilizer which avoids particle aggregation and provides stability during the synthesis. It has been reported that the rapid nanoparticle formation follows the Marangoni effect, which is associated with interfacial tension between the interface of solvent and non-solvent. This drug-entrapped nanoparticle formation is ruled by a combination of phenomena including flow, diffusion of solvent and variations in surface tensions [22,23,24]. During the nanoparticle synthesis, the solution is subjected to overnight stirring under room temperature conditions, so that the remaining acetone could be evaporated. Further, the polymeric solution was sonicated at low frequency to obtain stable, monodispersed nanoparticles [25]. A co-delivery system, based on the concept of combination chemotherapy has been prepared to improve the efficacy of therapeutic drugs against cancer. Size and size distribution of the polymeric nanoparticles were characterized by dynamic light scattering technique (Figure 2, Table 1). Also, the surface morphology was confirmed through scanning probe microscopy (AFM). The average size of the formed nanoparticles were in the range of 100–150 nm, which is considered satisfactory for effective drug accumulation in the tumour cells through the enhanced permeability and retention (EPR) effect [26]. Studies have shown that nanoparticles with a range of 200 nm generally extravasate in the tumour cells through vascular fenestrations and are able to escape reticuloendothelial system (RES) [27]. Also, the polydispersity of PLGA-PEI-EPI-PTX NPs was found to be 0.22, suggesting homogenous size distribution among the suspension. The results of particle size obtained through DLS are slightly larger as compared to AFM, as DLS measures the hydrodynamic size while the samples in AFM are deposited as a thin film in dried form. Moreover, the zeta potential of the modified nanoparticle was 41.95 ± 0.65 mV, which depicts an increased probability of the membrane particle interaction, as cell membrane is negatively charged.

**Figure 3:**
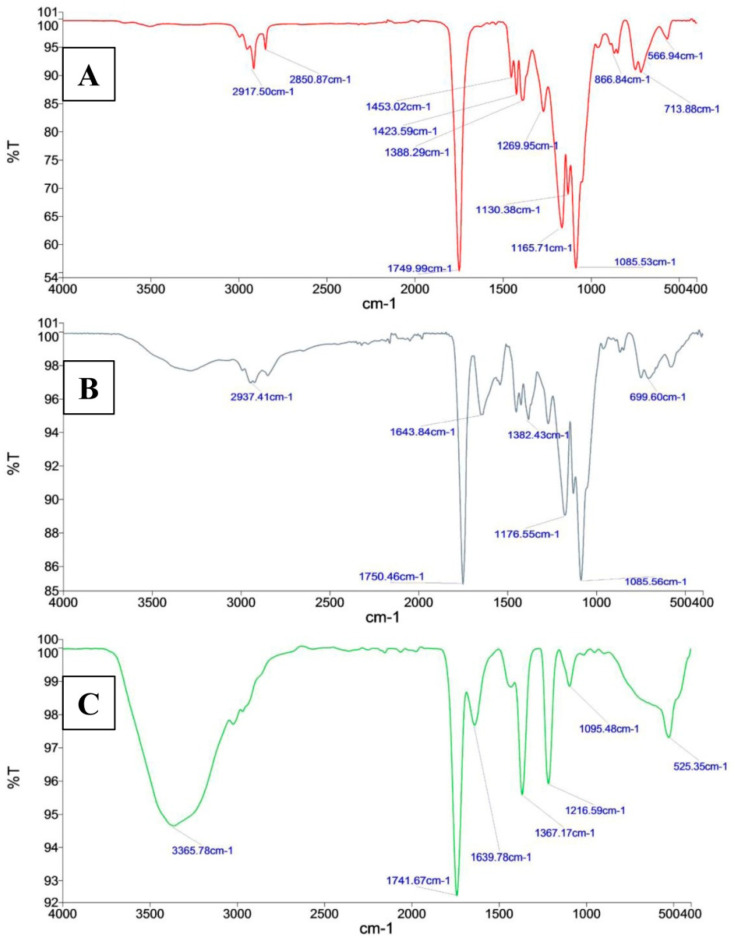
FTIR spectra of (**A**) void PLGA NPs, and (**B**) PLGA-PEI NPs and (**C**) PLGA-PEI-EPI-PTX NPs.

In the FTIR spectrum of PLGA and PLGA-PEI NPs, C-O carboxylic acid group stretching generated strong absorption peaks at 1165.71 cm^−1^ and 1176.55 cm^−1^, The C=O ester group had absorptions at 1749.99 cm^−1^ and 1750.46 cm^−1^ (Figure 3). These peaks were shifted in the FTIR spectrum of PLGA-PEI-EPI-PTX NPs to 1216.59 cm^−1^ for C-O carboxylic group stretching and 1741.67 cm^−1^ for the C=O ester group. These results indicated the interaction of chemical moieties present in PLGA and PEI with EPI and PTX, which consequently shifted the absorption peaks of C=O and C-O bonds. In addition, due to crosslinking of the carboxylic groups of PLGA with the amine groups of PEI, a secondary amide C=O absorption peak was observed at 1643.84 cm^−1^ and 1639.78 cm^−1^ in PLGA-PEI NPs and PLGA-PEI-EPI-PTX NPs, respectively. A broad peak at 2937.41 cm^−1^ and 3365.78 cm^−1^ are attributed to N-H bending vibrations of the amide groups in both the formulated nanoparticles i.e., PLGA-PEI NPs and PLGA-PEI-EPI-PTX NPs.

### 2.3. Encapsulation and Loading Efficiency

The encapsulation efficiency and drug loading content are important factors to enhance the effectiveness of therapeutic drugs, and to further analyse their applications at a clinical level. The amount of drug encapsulated in the modified nanoparticles was estimated through an indirect method. The absorbance of the free EPI and PTX were determined at wavelengths of 485 nm and 227 nm, respectively. The EE% and LE% of nanoparticles were in the range of 57.27% to 90.88% and 2.11% to 10.88%, correspondingly (Table 2). It was observed that the EE% of PTX was higher than that of EPI. This might be due to the fact that PTX, being hydrophobic in nature, easily interacts with the hydrophobic moiety of PLGA, while EPI associated with HCl form becomes hydrophilic and may leak out in the aqueous phase during the emulsification process [28]. Also, it was observed that PF68 concentration also influences the encapsulation and loading efficiency of the nanoparticles. The particles were prepared at varying concentration of 0.1–1% PF68 during the synthesis process. The maximum EE% and LE% for EPI and PTX with optimum size of nanoparticles were obtained at 0.4% PF68. Therefore, further studies were performed at this optimum PF68 concentration. The reason for low encapsulation at lower concentration of PF68 could be attributed to less chain in core and corona of the nanoparticles which was not optimal for the entrapment of drug [29]. Additionally, it was observed that the particles aggregate on increasing the surfactant beyond the critical concentration, and results in an increased particle size and instability [30]. Wang et al. reported that DOX-PTX nanostructured lipid carrier (NLC) possessed 82% and 84% of EE% for PTX and DOX. These obtained NLC were stable and facilitated the delivery to negatively charged cancer cells [31]. The results obtained are in accordance with the previous studies conducted against various cancer cell lines.

### 2.4. In Vitro Drug Release Studies

**Figure 4:**
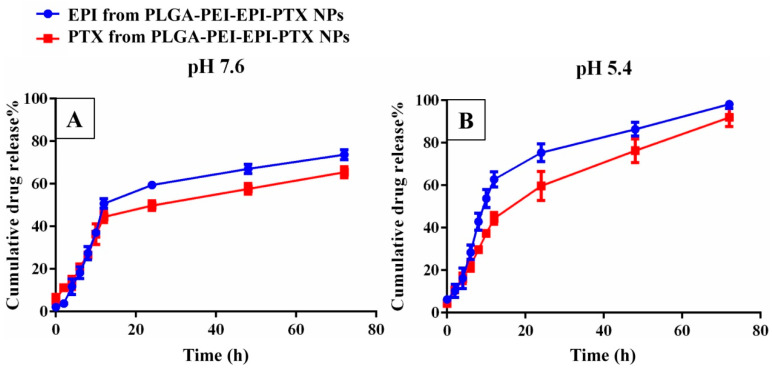
In vitro drug release study of epirubicin and paclitaxel from PLGA-PEI-EPI-PTX NPs at (**A**) pH 7.5 and (**B**) pH 5.4.

The in vitro cumulative drug release of EPI and PTX from PLGA-PEI-EPI-PTX NPs was studied at different pH values after definite time intervals (Figure 4). A sustained release pattern was observed in case of modified polymeric nanoparticles showing maximum drug release at pH 5.4 with 96.76% of EPI and 94.96% of PTX till 72 h. The slow release of drug was attributed to the diffusion of drugs (EPI and PTX) from the core of the nanoparticles and the breakdown of PLGA into its monomeric units of lactide and glycolic acids [32]. Also, the sustained release of the drug could be associated with the cross linking of lactic and glycolic acid moieties with the drug, resulting in the formation of a polymeric layer around the drug extending its release from the nanoparticles [33]. The results were consistent with previously reported studies in the literature. It was observed that the drug release was slower in pegylated liposomal formulations of ES-SSL-EPI/PTX during a 48 h time period. It was observed that the presence of PEG resulted in lower drug release [21].

To analyse the mode of drug release from the nanoparticles, various drug release kinetic models were applied including zero order, first order, the Higuchi model and the Korsmeyer-Peppas model. The drug release from PLGA-PEI-EPI-PTX NPs was best explained by the Higuchi model as it showed the maximum linearity (r^2^ = 0.9726), followed by Korsmeyer-Peppas (r^2^ = 0.966), First order (r^2^ = 0.954) and zero order (r^2^ = 0.8802) for PTX release. While for EPI, the release mechanism was best explained by first order (r^2^ = 0.965), Korsmeyer-Peppas (r^2^ = 0.9055), Higuchi model (r^2^ = 0.9039) and zero order (r^2^ = 0.7617) models, respectively. This suggested that the drug release followed a diffusion-controlled pattern from the matrix of the polymeric nanoparticle. Venugopal et al. showed similar drug release pattern for PTX following Higuchi model for drug release. It was observed that the drug release and kinetics was independent of anti-EGFR coupling [25].

### 2.5. Microscopic Analysis

#### 2.5.1. Cellular Uptake Analysis

**Figure 5:**
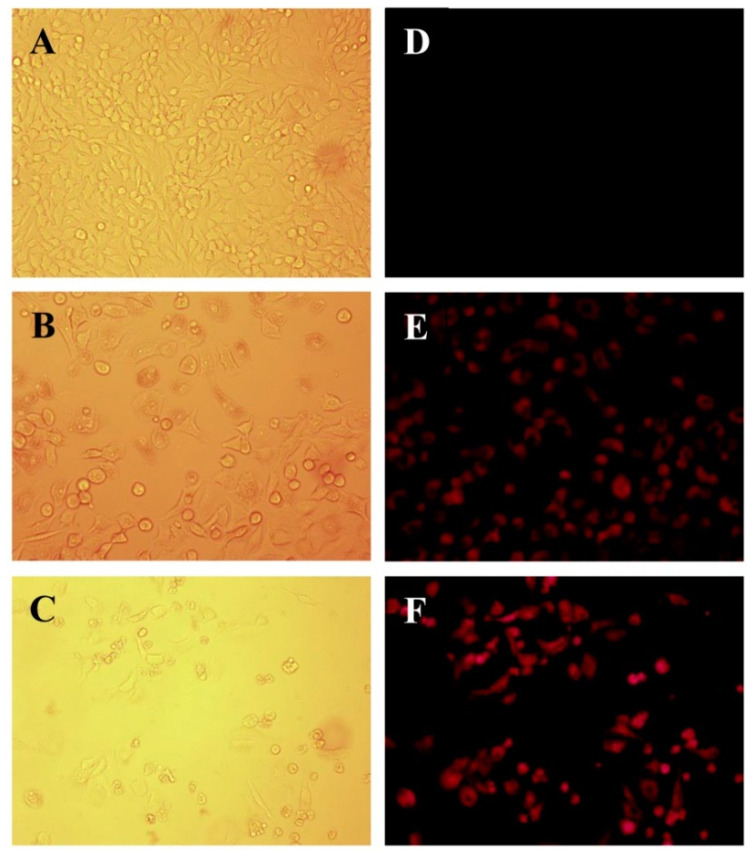
Microscopic images of cellular uptake in A549 cells, Bright field (left panel, A–C) and Fluorescence images (Right panel, D–F) of (**A**,**D**) untreated cells, (**B**,**E**) free EPI-PTX, and (**C**,**F**) PLGA-PEI-EPI-PTX NPs respectively.

EPI has an intrinsic fluorescence property which allows one to conveniently monitor the uptake of the nanoparticles along with the naïve drug within the cells, so cells were treated with their respective drug-loaded formulations and then visualized under a fluorescence microscope to investigate the relative amount of EPI taken up by the tumour cells. Strong fluorescent signals of EPI were observed from the cells treated with PLGA-PEI-EPI-PTX NPs formulation as compared to the free EPI-PTX drug combination, indicating higher uptake of the drug in the cells through the nanoparticles (Figure 5). Earlier reports indicated similar results in cervical carcinoma cells (HeLa) and suggested that nanoparticles facilitate the uptake of EPI in the cells as compared with the free drug [34]. Tang et al. reported that PEG modified estrogen (ES) targeted lipid based nanocarrier (ES-SSL-EPI) displayed stronger fluorescence in MCF-7 cells as compared to free EPI and SSL-EPI. It was observed that the fluorescence intensity increased to 1.75 fold times higher as compared to SSL-EPI. The results suggested that the nanoparticle mediated drug uptake is higher as compared to the naïve form [21].

#### 2.5.2. Acridine Orange/Ethidium Bromide Staining

**Figure 6:**
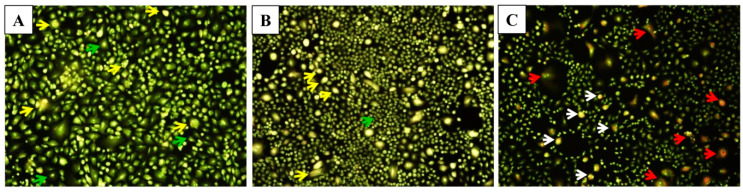
Microscopic images of A549 cells for apoptosis study. (**A**) Untreated A549 cells demonstrated normal structure (green arrows), early apoptosis (yellow arrows) such as chromatin condensation, cell membrane blebbing were observed after treatment with, (**B**) Free EPI-PTX and, late apoptosis (white arrows) and necrosis (red arrows) were noticed after treatment with, and (**C**) PLGA-PEI-EPI-PTX NPs, respectively.

The hallmarks of cells undergoing apoptosis include cell shrinkage and rounding, surface membrane blebbing, chromatin condensation followed by nuclear fragmentation and cellular disassembly [35]. For the morphological analysis, selective fluorescent DNA binding dyes (such as acridine orange/ethidium bromide) methods are preferred owing to their simplicity, accuracy and rapidity. Such assays eliminate cell fixation step, thus eluding several potential artefacts [36]. In the present study, the induction of apoptosis after the treatment with modified nanoparticles determined by fluorescence staining using a DNA-specific dye. Acridine orange/ethidium bromide (AO/EtBr) staining was performed to investigate necrosis, chromatin condensation in the cells. Acridine orange can permeate the normal cell membrane, the cells were observed with green fluorescence. While yellow or orange coloured bodies were observed in apoptotic cells and apoptotic bodies which were formed due to nucleus shrinkage, chromatin condensation. Lastly, necrotic cells appeared as red coloured fluorescence owing to their loss in membrane integrity [37]. This dual staining facilitates to easily differentiate between early apoptotic, late apoptotic and dead cells, and also to detect mild DNA injuries [38]. In the present study, EPI-PTX combination drug and nanoparticles induce cell membrane blebbing, and chromatin condensation, indicating early apoptosis (Figure 6). While necrosis was observed in the cells treated with the PEI modified nanoparticles. The obtained results are in accordance with the previous studies. As shown earlier, the combined treatment with PTX caused higher apoptotic effect as compared with the individual drugs. Sharma et al. demonstrated that PTX in combination showed higher percentage of apoptotic cells when administered in MCF-7, MDA-MB 231 (human breast cancer cells) [39]. Also, in prostate cancer, it was shown that on treatment with combination drugs, there was a significant decrease in BCl-2 mRNA expression, and an increase in Bax (*p* < 0.05). The results further validated the nuclear irregularities and apoptosis which were observed microscopically.

#### 2.5.3. Wound Healing Assay

**Figure 7:**
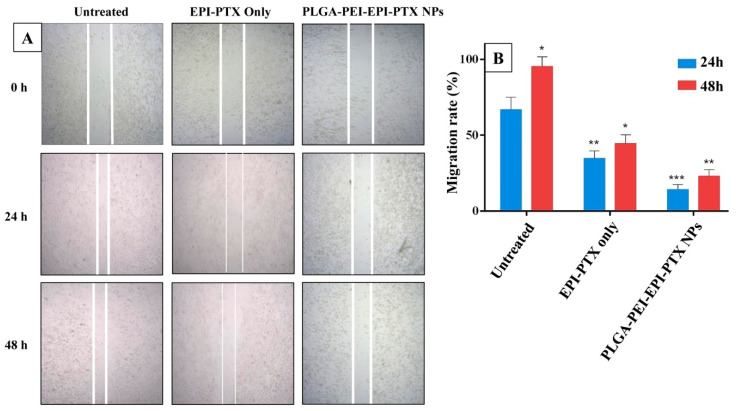
Scratch assay. (**A**) Microscopic images of A549 cells showing reduction of scratch at different time points, and (**B**) Graphical representation of the scratch at 24 h and 48 h. * *p* < 0.05, ** *p* < 0.001, *** *p* < 0.0001.

Cell migration and invasion play a crucial role in the progression of the disease. To evaluate the effect of formulated nanoparticles on cell migration, a scratch type wound healing assay was performed. The process of wound closure in the scratched area through migratory cells was monitored through inverted microscopy after 24 and 48 h (Figure 7). As revealed from the results, the cell migration after 24 h was decreased to 79.08% when PLGA-PEI-EPI-PTX NPs were administered, while this value was 46.65% when given in combination form. There was therefore a significant (*p* < 0.0001) decrease in the migration of cancer cells in the nanoparticle treated wells, as compared with both the control and naïve drug mixture. The reason could be that the PEI-modified nanoparticles could enter more easily into the cells and resulted in higher intracellular concentration, which in turn affected the migration ability of the cells. PTX is known to disrupt the formation of lamellipodia, by restricting the number of plus end microtubules. This inhibits the microtubule dynamics and thereby the migration of cell [40]. Similarly, EPI has been reported to suppress the expression of vascular endothelial growth factor (VEGF) and matrix metalloproteinase-9 (MMP-9), which are the key players in stimulating invasion and angiogenesis in various type of cancer [41]. Thus, the combination with different mechanism markedly inhibited the cell migration even at lower concentration of the drugs. The obtained results suggested that the nanoparticle formulated combinational therapy is more effective in reducing the cell migration and invasion as compared with the free agents.

### 2.6. In Vitro Cytotoxicity Studies

**Figure 8:**
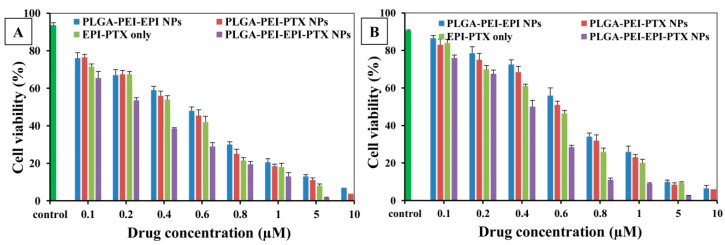
Cell viability as determined by MTT assay. The % cell viability of A549 cells treated with PLGA-PEI-EPI NPs, PLGA-PEI-PTX NPs, EPI-PTX only and PLGA-PEI-EPI-PTX NPs, at (**A**) 24 h time point, (**B**) 48 h time point. Data are shown as mean ± SD (n = 3).

**Figure 9:**
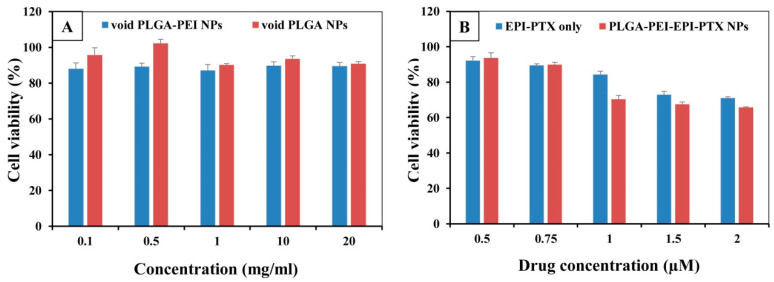
Cell viability in HEK 293 normal cell lines as determined through MTT assay. The cells were treated with (**A**) void PLGA nanoparticles and with (**B**) PLGA-PEI-EPI-PTX NPs. Data are shown as mean ± SD (n = 3).

The cell viability assays were performed to analyse the biocompatibility and anticancer activity of nanoparticles and their potential was compared with the combined drugs. EPI and PTX were loaded in the same polymeric delivery system to demonstrate the synergistic effect of these two therapeutic drugs (Figure 8). The IC_50_ value of EPI and PTX was significantly reduced to 0.543 µM in the PLGA-PEI-EPI-PTX NPs form as compared with either the mixture or with individual nanoparticles. Also, it was demonstrated that the cytotoxicity was increased to 4.5-fold for PTX and 3.9-fold for EPI when administered in combination through formulated nanoparticles. The results showed that the anticancer activity of the modified nanoparticles was concentration dependent. The enhanced efficacy and higher toxicity of the formulated particles can be attributed to the increased synergistic effect of the drugs in nanoparticles. The PEI present on the nanoparticles results in an increase uptake of the drug loaded nanoparticles by the cell [42]. This in turn resulted in an increase efficacy of the therapeutic molecule in the cancer cells. Similarly, Tang et al. reported that the IC_50_ value of PTX and EPI was reduced when given in combination against breast cancer cells [21]. In another study, PTX was administered with doxorubicin (DOX) against NCL-H640 cells (lung cancer cells). Results dictated a considerable increase (*p* < 0.05) in their cytotoxic effect as compared with individual drug nanocarriers (three times), or with free drug (nine times) [31]. Further, for the evaluation of biosafety of the nanoparticles on normal cell lines, we treated the formulated nanoparticles with widely available HEK 293 normal human cell lines (Figure 9). It was observed that the particles showed no significant toxicity in HEK 293 cell lines. These results were in agreement with the previous studies mentioned in the literature [42,43].

### 2.7. Western Blotting

**Figure 10:**
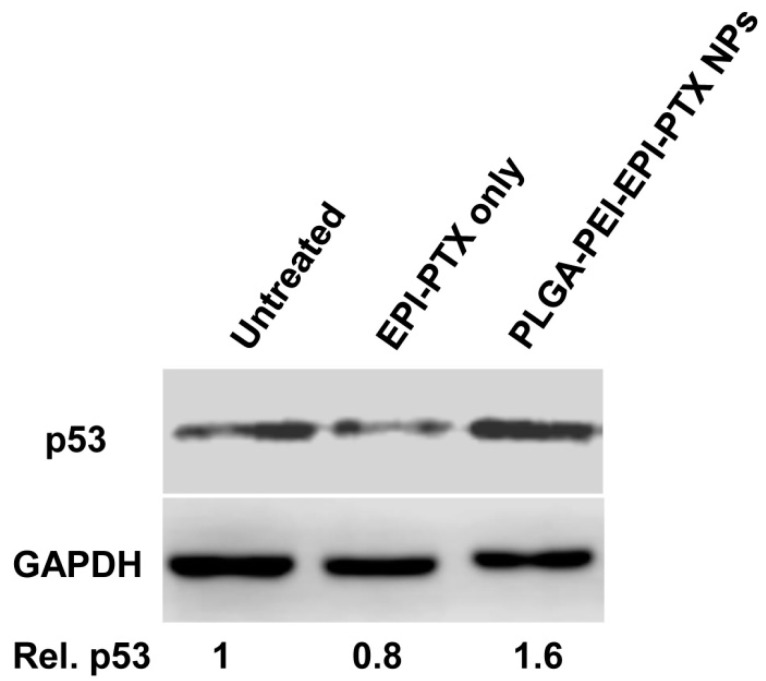
Western blot analysis study: A549 cells treated with EPI-PTX only and PLGA-PEI-EPI-PTX NPs, showing overexpression of p53 relative to control, and normalized protein expression. GAPDH was used as a loading control. The experiments were conducted in three replicates.

Tumour suppressor gene p53 acts as a key regulator to maintain the cellular apoptotic activity, while any failure in this process results in tumour progression. The p53 gene induced apoptosis causes cell death either following transcription dependent or independent pathways. Moreover, this process gets activated under hypoxic condition, DNA damage, aberrant oncogene expression, DNA repair, apoptosis and many more [44]. Additionally, p53-mediated apoptosis has been shown to contribute in chemotherapy-associated cell death [45,46], so in the current study, western blot analyses were performed to show the effect of p53 expression when administered nanoparticle formulation. The modifying effect of PEI over PLGA nanoparticles resulted in an increase in cellular uptake as compared with the free EPI and PTX. It was evident from the western blot results that EPI and PTX in combination stimulate the expression of p53 levels (Figure 10). After A549 cells were treated with EPI and PTX in combination as well as in nanoparticle formulation, the expression levels p53 protein was gradually increased (Figure 10). This showed an effective drug delivery of EPI and PTX into the cells as compared to the free drug. Furthermore, it was evident that the drug triggers DNA damage by stimulating p53 expression levels [47]. Millour et al. investigated the effect of p53 on Forkhead box M1 (FOXM1) expression after EPI treatment in MCF-7 cells. It was observed that EPI suppressed the FOXM1 expression through the activation of p53, and leads to the induction of apoptosis in cancer cells [48]. Additionally, EPI liposomes showed to suppress the expression of PI3K, MMP-2, MMP-9, and induced apoptotic enzyme caspase 3 in non-small cell lung cancer [49]. Moreover, p53 induced the expression of COX-2 in H460 and A549 cells when administered with PTX. It was shown that p53 expression could alter the tumor sensitivity towards chemotherapy through the induction of COX-2 [49]. This study validated the synergistic effect of nanoparticles in the animal model cell lines, yet not been investigated in vivo. It is deduced that in the future studies, their synergistic effect can further be validated in various pre-clinical and clinical levels.

## 3. Materials and Methods

### 3.1. Materials

PLGA (50:50 7 kDa–17 kDa, acid terminated), polyethylenimine (25 kDa, branched), epirubicin and paclitaxel were purchased from Sigma Aldrich (Darmstadt, Germany). Acetone was purchased from Sisco Research Laboratories Pvt. Ltd. (Mumbai, India). PF68 surfactant, Alamar blue, PBS buffer and sodium bicarbonate were procured from HiMedia (Mumbai, India). Dulbecco’s modified Eagle’s medium (DMEM), fetal bovine serum (FBS) were obtained from Gibco Life Technologies (Carlsbad, CA, USA). In-house distilled water was used during the studies.

### 3.2. Evaluation of Combination Ratio of EPI and PTX

#### 3.2.1. Determination of Cell Toxicity

The toxicity of PTX and EPI were administered individually as well as in combination in A549 cancer cell line using an Alamar Blue assay. Cells were plated in 96 well plate at a density of 8 × 10^3^ cells/well. The cells were allowed to adhere to the plate surface by incubating it overnight at 37 °C temperature in 5% CO_2_ concentration and 90% relative humidity. Following overnight incubation, the chemotherapeutic drugs were added individually and in combination at five fixed EPI: PTX molar ratios (1:5, 1:3, 1:1 3:1, 5:1) to the cells. After 48 hr incubation, cell viability was determined using Alamar Blue. The results were assessed by measuring the absorbance at 570 nm and 600 nm. The concentration at which the dose resulted in 50% cell inhibition (IC_50_) was analyzed by employing the Hill equation with best fit (GraphPad Prism software, GraphPad Inc., San Diego, CA, USA). The IC_50_ was calculated from three individual experiments.

#### 3.2.2. Determination of Combination Index Values

The combination of EPI and PTX was calculated through median effect analysis, originally established by Chou and Talalay. Using this method, combination index (CI) is determined from growth inhibition curves. For this, the IC_50_ values for both the individual as well as combination drugs are need to be calculated. The CI of the two drugs was estimated through the following Equation (1):

(1) $$\mathrm{CI}=\frac{d_{ptx}}{D_{ptx}}+\frac{d_{epi}}{D_{epi}}$$

where *d_ptx_* and *d_epi_* were the doses of paclitaxel and EPI in the combination which kills α% cells, *D_ptx_* and *D_epi_* are the doses of drug which kills α% when given as monotherapy. Based upon the CI index, the combination of the drugs can be grouped as additive effect with CI = 1, synergistic CI < 1 and antagonistic effect with CI > 1. CI values were calculated at various fraction affected (Fa) of the cells. The Fa value ranges from 0–1, where Fa = 0 means 100% viable cells and Fa = 1 means 0% viable cells.

### 3.3. Preparation of Epirubicin Paclitaxel PLGA PEI Nanoparticles

PLGA-PEI nanoparticles entrapping EPI and PTX (PLGA-PEI-EPI-PTX NPs) were prepared through a solvent displacement technique. Briefly, for void PLGA-PEI nanoparticles, organic and aqueous phases were prepared separately. For the organic phase, 10 mg of PLGA and 4 mg of PEI were mixed in acetone and was kept on a magnetic stirrer (UC152 hot plate stirrer, Stuart, St. Neots, UK) for 15 min. This was further added dropwise to aqueous solution of 0.4% PF68 under continuous stirring at 500 rpm for 8–12 h to remove the organic solvent from the solution. Similarly, PLGA-PEI-EPI-PTX NPs were prepared by adding different molar concentration of EPI and PTX alone and in combinations (1:5, 1:3, 1:1 3:1, 5:1) in the organic phase containing PLGA and PEI polymer. Afterwards, these were added in dropwise manner into aqueous phase. Further, the organic phase was allowed to evaporate and centrifuged at 16,000× *g* for 20 min at 4 °C (Heraeus, Fresco 17, Thermo Scientific, Waltham, MA, USA). The obtained nanoparticles were suspended in double distilled water. These were washed thrice and supernatant was kept in separate vial and further used for drug quantification analysis. The nanoparticles were freeze dried and stored at 4 °C until further use.

### 3.4. Physicochemical Characterization of the Nanoparticles

The zeta potential and size distribution of the PLGA-PEI-EPI-PTX NPs were measured using dynamic light scattering (DLS) (Zetasizer Nano Series, Malvern PanAnalytical, Malvern, UK). The particles were dispersed in MilliQ water and were further employed for size analysis at 5 mW HeNe laser of 633 nm wavelength, with scattering light at 173° angle. In addition, an Innova AFM multimode scanning probe microscope (Bruker, Billerica, MA, USA) was employed for the analysis of surface morphology of the nanoparticles. It was carried out by placing a drop of diluted suspension of nanoparticles onto the glass slide. It was dried under vacuum at 25 °C, and 15 bar pressure for a period of ~12 h. Further, AFM imaging was performed with tapping mode at resonance frequencies of 260–340 kHz. Moreover, the involvement of functional group between the interactions of PEI with PLGA polymer was assessed by Fourier transformed infrared spectroscopy (FTIR) analyses. The lyophilized samples were pelleted with KBr and scanned against the blank KBr pellet in the range of 4000–450 cm^−1^. The data obtained revealed the presence of possible functional moiety in the modified nanoparticles. Drug loading and in-vitro drug release studies of the PLGA-PEI-EPI-PTX NPs were determined through UV-vis spectrophotometer (Evolution 201, Thermo Scientific, Waltham, MA, USA) at their respective optimum wavelengths.

### 3.5. Encapsulation and Drug Loading Efficiency

The drug encapsulation (EE) and loading efficiency (LE) of the dual-drug loaded nanoparticles was determined spectrophotometrically. To enumerate % LE, specific weighed amount of drug loaded nanoparticles (10 mg/10 mL) were dissolved in DMSO. The PTX and EPI concentration was then assessed by absorbance measurement using standard curve of EPI and PTX absorbance (at wavelengths of 485 nm and 227 nm, respectively) against different known concentrations of the drug, respectively (Equation (2)):

(2) $$Loading\;efficiency\;\left(\%\right)=\frac{amount\;of\;the\;drug\;in\;NPs\;\left(mg\right)}{weight\;of\;the\;drug\;loaded\;NPs\;\left(mg\right)}\times 100$$

Encapsulation efficiency (% EE) was analyzed through centrifugation technique. The formulated NPs were pelleted through centrifugation at 17,000× *g* for 20 min at 4 °C. The pellet was further washed and stored at 4 °C until use and the supernatant was measured spectrophotometrically for free drug. The amount of free drug was estimated through the calibration curve of drug solutions with known concentrations at their particular wavelengths (Equation (3)):

(3) $$Encapsulation\;efficiency\;\left(\%\right)=\frac{amount\;of\;the\;drug\;encapsulated\;in\;NPs\;\left(mg\right)}{initial\;amount\;of\;drug\;used\;during\;synthesis\;\left(mg\right)}\times 100$$

### 3.6. In Vitro Drug Release Kinetics

The PLGA-PEI-EPI-PTX NPs were dispersed in phosphate buffer (pH 5 and 7.4) at a final concentration of 1 mg/mL and kept in a shaker at 37 °C. The samples were drawn out at different time intervals i.e., after 2, 4, 6, 8, 10, 12, 24, 48 and 72 h. The supernatant of the samples were collected after centrifugation at 17,000× *g* for 15 min. The volume was compensated with an equal volume of fresh buffer medium. The supernatant contains the released PTX and EPI which was further measured through UV-vis spectrophotometry. The absorbance was recorded at 227 nm and 485 nm wavelength, and cumulative drug release profile was analysed as per the obtained absorbance. Additionally, to enumerate the release mechanism and kinetics from the nanoparticles, the results were fitted in various kinetic models, to get the best fit.

### 3.7. Cell Culture Studies

Human alveolar lung carcinoma, A549 cell line was procured from the National Centre for Cell Science (NCCS, Pune, India). The cells were grown in DMEM culture medium supplemented with 10% fetal bovine serum (FBS), 100 U/mL penicillin-streptomycin. The cells were continuously passage when reached confluency. These were maintained at 5% CO_2_, 37 °C temperature and with 5% humidity.

### 3.8. Microscopic Analysis

#### 3.8.1. Cellular Uptake Study

A549 cells (at a density of 8 × 10^3^ per well) were cultivated in 96 well plate. The plate was kept in incubator for 24 h to allow adherence of cells. The media was replaced with fresh media containing EPI and PTX in combination, PLGA-PEI-EPI-PTX NPs at their IC_50_ concentration and the cells were incubated for 24 h at 37 °C. After incubation, cells were washed with PBS 3 times, to remove any excessive nanoparticles that were not taken up by the cells. The cellular uptake was monitored through fluorescence microscope (DMI 6000 B, Leica Microsystems, Wetzlar, Germany) and the images were captured by a digital camera.

#### 3.8.2. Apoptotic Analysis through Acridine Orange/Ethidium Bromide Staining

The apoptotic characteristics like nuclear condensation, cell membrane blebbing, and necrosis can be observed under fluorescence microscope after staining with DNA specific dye. Briefly, 8 × 10^3^ cells per well were seeded in a 96 well plate. After 24 h incubation, wells were treated with media containing EPI and PTX in combination, PLGA-PEI-EPI-PTX NPs for 24 h at 37 °C, 5% CO_2_. The wells were washed 3 times with PBS. Further, the cells were stained with acridine orange (AO; 100 µg/mL) and ethidium bromide (EtBr; 100 µg/mL) at 1:1 ratio. After staining for 3–5 min, cells were washed with PBS and finally suspended in PBS for microscopic observation. The cells were examined under fluorescence microscope and their images were captured [49].

#### 3.8.3. Cell Migration Assay

The effect of drug entrapped nanoparticles on the migration of cancer cells was analysed through in vitro scratch wound healing method. The cells were seeded in a 24 well plate with a cell density of 1 × 10^5^ cells per well. The cells were kept in CO_2_ incubator for overnight to allow to grown to full confluency. The cells monolayer was crossed with a sterile 20 µL pipette tip in the centre of each well. The wells were washed with PBS 3 times, to remove the scraped cells from the wells. The cells were further administered with combination of EPI-PTX only and PLGA-PEI-EPI-PTX NPs to the wells. After incubation for 24, 48 h, the media was replaced with PBS, the wound gap was observed under an inverted microscope (Primovert, Zeiss, Oberkochen, Germany) and the image was taken using a Zeiss camera. The software used for the image acquisition was Zen2 elite. The migration rate was calculated using the following Equation (4):

(4) $$Migration\;rate\;\%=\frac{\left(Initial\;scratch\;width-scratch\;width\;after\;time\;t\right)}{Initial\;Scratch\;width}\times 100$$

### 3.9. In Vitro Cytotoxicity Studies

Cell viability of the formulated nanoparticles was determined through an alamar blue assay. Briefly, A549 cells were seeded in 96 micro-well plates at a cell density of 8 × 10^3^ cells per 100 µL. The cells were kept in an incubator with 5% CO_2_, 37 °C for overnight, so that cells get adhered to the plate surface. Further, each well was administered with different concentrations of nanoparticles and drugs along with control (cells in complete media containing FBS and DMEM). After incubation of 24 and 48 h, the cells were analysed for cell viability through the Alamar Blue technique. For this, the cells were initially replaced with fresh medium and then 10% of resazurin (0.15 mg/mL in PBS) was added to it. The wells were allowed to incubate for 2–4 h in an incubator and later the growth of the cells were measured through multi-well plate ELISA reader (Thermo Multiskan GO, Thermo Scientific, Vantaa, Finland). The change in the absorbance was recorded at 570 nm and 600 nm respectively. Percentage cell viability was calculated against the control cells.

### 3.10. Western Blot

Western blots were performed to determine the effect of p53 levels after the treatment of nanoparticles. Total cell lysate was separated and collected after the treatment using RIPA lysis buffer (50 mM Tris-HCl, pH 8.0, 1 mM EDTA, 150 mM NaCl, 1% Nonidet P-40) containing freshly added protease inhibitor cocktail (Thermo Scientific). Concentration of proteins was quantified through Bradford assay (HiMedia). Then samples were denatured and equal amount of protein (40 µg/well) was loaded onto sodium dodecyl sulphate (SDS) polyacrylamide gel (PAGE) (BioRad, Richmond, CA, USA) for electrophoresis. Proteins were transferred to nitrocellulose membrane (NCM) using electrophoretic transfer (BioRad). To avoid nonspecific reactivity, blot was incubated for 1 h at room temperature in Tris-buffered saline-Tween 20 (TBST) buffer with 3% bovine serum albumin (BSA). Blot was incubated overnight at 4°C with primary antibodies of p53 (sc-47698, dilution, 1:500) and GAPDH (sc-59540, dilution, 1:2500) all purchased from Santa Cruz Biotechnology (Dallas, TX, USA). Subsequently, the blot was further incubated with secondary antibody m-IgG BP-HRP (sc-516102, dilution 1:5000) and visualized with enhanced chemiluminescence detection system Li-COR C-digit Blot scanner (LI-COR Biosciences, Lincoln, NE, USA).

### 3.11. Statistical Analysis

All the experimental studies were performed in triplicates. The data represented the mean ± standard deviation (S.D.) values and statistical analysis was accomplished with GraphPad Prism (version 6.01, GraphPad Software Inc., San Diego, CA, USA). The treated groups were compared with the control by the application of Student *t*-test and analysis of variance (ANOVA). The data were considered statistically significant at a *p* value of <0.05.

## 4. Conclusions

In summary, the present work demonstrated the design and development of uniform PEI-coated PLGA nanoparticles for the dual delivery of EPI and PTX in a A549 lung carcinoma cell line. The drug release from the nanoparticles was more at an acidic pH of 5.4 as compared to a basic pH of 7.6. This may be attributed to the cleavage of ester bond present between the monomeric units of lactic and glycolic acids. The drug release followed a sustained diffusion from the matrix. The synergistic effect of EPI with PTX in nanoparticles resulted in an increase in cell death as compared to the naïve drugs. Moreover, the combinational approach was also effective against inhibition of cell migration and invasion of cancer cells.

## Figures and Tables

**Table 1:** Represents the nanoparticles size, polydispersity index and zeta potential. Data are shown as mean ± SD (n = 3).

Formulations	Size (nm)	PDI	Zeta Potential (mV)
PLGA NPs	101.59 ± 8.87	0.16 ± 0.04	−29.06 ± 1.015
PLGA-EPI-NPs	117.05 ± 5.91	0.20 ± 0.06	−24.5 ± 1.1
PLGA-PTX NPs	139.70 ± 1.73	0.19 ± 0.002	−21.25 ± 0.55
PLGA-PEI-EPI-PTX-NPs	240.93 ± 12.70	0.27 ± 0.08	41.95 ± 0.65

**Table 2:** Represents the encapsulation and loading efficiency of nanoparticles. Data are shown as mean ± SD (n = 3).

S.No.	Formulations	Ratio EPI: PTX	% Encapsulation Efficiency		% Loading Efficiency	
			Epirubicin	Paclitaxel	Epirubicin	Paclitaxel
1	PLGA-EPI NPs	-	74.09 ± 0.42	-	9.56 ± 0.11	-
2	PLGA-PTX NPs	-	-	90.88 ± 1.73	-	10.88 ± 0.67
3	PLGA-PEI-EPI-PTX-NPs	1:5	74.89 ± 5.92	87.25 ± 5.67	9.58 ± 3.56	6.99 ± 1.24
4	PLGA-PEI-EPI-PTX-NPs	1:3	67.75 ± 0.41	77.05 ± 8.80	6.99 ± 1.54	7.95 ± 3.32
5	PLGA-PEI-EPI-PTX-NPs	1:1	77.34 ± 2.99	84.61 ± 0.13	5.35 ± 2.19	7.26 ± 0.33
6	PLGA-PEI-EPI-PTX-NPs	3:1	57.27 ± 2.43	75.20 ± 0.86	4.92 ± 0.27	3.31 ± 0.40
7	PLGA-PEI-EPI-PTX-NPs	5:1	59.66 ± 5.28	62.94 ± 8.0	5.61 ± 1.02	2.11 ± 0.12
